# Struggle and banality of belonging to Europe. Cultural Europeanization from the perspective of the Central and East European citizens

**DOI:** 10.1080/14782804.2023.2207000

**Published:** 2023-04-28

**Authors:** Katja Mäkinen, Sigrid Kaasik-Krogerus

**Affiliations:** a Department of Music, Arts and Culture Studies, University of Jyväskylä, Jyvaskyla, Finland; b Department of Finnish, Finno-Ugrian and Scandinavian Studies, University of Helsinki, Helsinki, Finland

**Keywords:** Europeanization, belonging, identity, ethnography, cultural heritage

## Abstract

The European Union (EU) has developed cultural policy initiatives that seek to promote cultural Europeanization with the purpose of constructing European identity narratives and facilitating citizens’ sense of belonging to Europe and the EU. The article focuses on the citizens’ perspective to cultural Europeanization through ethnographic research on one central action in the EU cultural policy, European Heritage Label (EHL). We analyse the interviews conducted in selected EHL sites with Central and East European (CEE) citizens who were visiting the sites as well as with cultural heritage practitioners working at three EHL sites located in CEE countries. We ask how the practitioners and the visitors engage with European identity narratives and elaborate their European belonging. We especially scrutinize how everyday encounters and experiences, such as mobility, shape identifications with ‘Europe’ and perceptions of what is ‘European’. The interviews are interpreted in the theoretical framework of ‘being’ and ‘becoming’ European. This framework indicates a centuries-long liminal position of the Central and Eastern Europe. It enables us to scrutinize CEE citizens’ sense of belonging to Europe in an intersection of dual Europeanization, i.e. cultural Europeanization and ‘Europeanization’ of the CEE countries to overcome this liminal position and become ‘true’ Europeans.

## Introduction

As a response to several transformations and challenges in Europe, the European Union (EU) has developed cultural policy initiatives to construct European identity narratives and facilitate citizens’ sense of belonging to Europe and the EU. The EU's flagship heritage action, the European Heritage Label (EHL), is one example of that. The main objectives of the action are ‘strengthening European citizens’ sense of belonging to the Union’ and ‘strengthening intercultural dialogue’ (EP&C European Parliament and the Council 2011, 3). The EHL was initiated as an official EU action in 2011 and by now the European Commission (EC) has awarded 60 sites with the Label.

EU’s cultural policy initiatives and their attempts to construct European identity and facilitate citizens’ sense of belonging have been scrutinized in the academia as cultural Europeanization (see Lähdesmäki et al. 2021, 1–2). Following the evolution of the EU integration, distinction between economic, political and cultural Europeanization is made in the research. Economic Europeanization refers to the launch of the current EU integration in the 1950s that started from the economic sector. Political Europeanization indicates widening of the integration to the political sphere, particularly since the Maastricht Treaty in 1992 but gradually already earlier. While both economic and political Europeanization contribute to forming European identity (e.g. Kantner 2006), constructing common identity is one of the main objectives of cultural Europeanization. Although the EU has paid a progressive attention to culture, cultural heritage and identity since the 1970s, scholars have positioned cultural Europeanization to the 1990s. The EU and its predecessors have used cultural activities for making the EU visible and feelable in citizens’ everyday life to construct the emerging EUropean community (Kraenzle and Mayr 2017, Karlsson 2010 (2014), 4; Lähdesmäki 2019). Making visible both the distinction and overlaps between Europe and the EU, the term EUrope(an) refers to the idea of Europe produced in and through EU initiatives (about the term, see Lähdesmäki et al. 2020, 14–15). As such, the term is closely linked to the idea of Europeanization ‘from above’, i.e. as conditioned by the EU. As an official action of the EU, the EHL is a prime example of the EU’s attempts of building a (cultural) EUrope from above but simultaneously from below, through micro-level experiences.

This article scrutinizes Europeanization through ethnographic research on the EHL. The analysis broadens our understanding of the Europeanization as a dynamic, multifaceted and multidirectional process in two ways. First, we focus on the citizens’ perspective to the EHL and explore identity constructions and sense of belonging to Europe from below. Based on the interviews with the visitors from Central and Eastern Europe (CEE) as well as practitioners working at three EHL sites located in CEE, we scrutinize how everyday encounters and experiences shape identifications with ‘Europe’ and perceptions of what is ‘European’. We ask what meanings the visitors and the practitioners of the EHL sites give to Europe and the European and how they construct their relations and identifications to Europe. While these citizens are not specific or different from other EU citizens (more or less ‘European’), the EHL sites provide them a context for reflecting the questions of Europeanization. Hence, scrutinizing their understandings can be framed as analyzing the EHL as cultural Europeanization from below (see also Lähdesmäki et al. 2021). This unveils how the goal of the EU’s cultural policy to produce a European identity – i.e. cultural Europeanization from above – is interpreted in the grassroot practices. Moreover, the analysis shows the potential contingency and contradictions of this process.

Second, the article brings together citizens’ everyday activities like visiting or working at heritage sites and the political context of multifaceted Europeanization. Our theoretical framework of ‘being’ and ‘becoming’ European indicates a centuries-long liminal position of the CEE countries (Mälksoo 2006). This framework enables us to scrutinize CEE citizens’ sense of belonging to Europe in an intersection of dual Europeanization, i.e. EHL sites as exemplifying cultural Europeanization that is lead by the EU but uses the grassroot-level activities and ‘Europeanization’ of the CEE countries to overcome this liminal position and become ‘true’ Europeans. We ask how the interviewees construct the positions of ‘being’ and ‘becoming’ European in relation to EUropean heritage on a daily basis and, as part of the same process, identify themselves in the framework of Europeanization by either struggling over European belonging or taking this belonging for granted and thereby developing a sense of banal belonging to Europe.

To introduce our framework, we will next discuss the processes of Europeanization as well as the idea of belonging as an approach to experiencing ‘Europe’. After that, we will briefly introduce the ethnography of Europeanization as a methodological innovation that enables to analyse Europeanization as a multifaceted process and investigate how notions of Europe and European identity and belonging are constructed from below. In the empirical analysis, we analyse the interviewees’ perceptions of Europe as well as their sense of belonging to it, constructed through their engagement with the EHL sites. The article ends with conclusions about the role of micro-level experiences in the processes of Europeanization.

## Being, becoming and belonging to Europe

In its widest sense, Europeanization can be understood as an integration constituted by several intertwined transnational processes in which ideas and practices transform because of a mutual interaction between numerous actors among European states and in particular, with the institutions, policies and regulations of the EU. Various scholars point out the institutional dimension of the Europeanization as a process of international socialization of national institutions and the impact it has on the legislation (Börzel and Risse 2003; Schimmelfennig 2000). Europeanization is depicted as an integration process where formal and informal rules, procedures, policies, and norms are constructed at the European scale and then diffused to domestic institutions (Radaelli 2000, 4; Schimmelfennig 1998, 198–200; Schimmelfennig 2000, 109–112). Scholars have also paid attention to the twofold character of Europeanization including the movement from the European scale to the national and subnational ones, as well as the other way round (e.g. Börzel 2002; Kaasik-Krogerus 2019b). Furthermore, in addition to the top-down process in which various institutions play a prominent role, scholars have underlined the importance of Europeanization from below (e.g. Kaiser, Krankenhagen, and Poehls 2014; della Porta 2020; Sassatelli 2008, 225; Weber 2021). This notion refers to the ways (civil) society as well as the citizens experience, engage with and/or challenge the ideas, practices and performances of Europeanization in their everyday life, e.g. through transnational activities or interpreting contemporary political transformations (Carpentier 2021, 235–237; Heidenreich 2019; Trenz 2014).

As a process aimed at integration and socialization, Europeanization is closely related to the notions of identity and belonging. Although complementary, these two are not synonymous. While identity is articulated in the stories and narratives of ‘being’ to express who we think we are and who are the Others, belonging is related to the identification with these stories, which underlines the questions of inclusion and exclusion. Identity narratives consist of the positions of self and Others, whereas belonging is about the experiences of access and participation related to these narratives (Anthias 2008, 8; see also Kaasik-Krogerus 2019a). ‘Banal nationalism’ (Billig 1995) and ‘banal Europeanism’ (Cram 2009b, 2012) highlight the importance of mundane aspects in the identification processes and refer to taking subject position of the national/European ‘us’ as part of everyday interaction. This process can be seen as a connecting link between identity as narratives and belonging as a sense of identification with these narratives (see also Kantner 2006, 509). Scholars have pointed out a normative impetus as well as a temporal aspect of the intensification of cultural Europeanization aimed at constructing European identity (e.g. Carpentier 2021 234, 237; Kaiser, Krankenhagen, and Poehls 2014). This makes Europeanization a process of becoming that is contradictory and contingent with multiple articulations of what constitutes ‘us’ and ‘Others’ (Delanty and Rumford 2005, 19).

This is even more true in the case of the CEE countries and their citizens in the context of the EU as a process of Europeanization. Especially during the EU Eastern Enlargement in the 1990s and the beginning of the 2000s, the accession process was elaborated in terms of simultaneous ‘being’ and ‘becoming’ European. On the one hand, the whole accession process was legitimized both in the CEE countries and inside the EU by referring to the candidates as ‘being’ European countries. In everyday public discussion, metaphors like ‘return to Europe’ and ‘family reunion’ made the ‘being’ explicit (Petersson and Hellström 2003; Kølvraa 2017, 13; Visvizi and Tokarski 2018, 114; Kaasik-Krogerus 2021). On the other hand, the fact that the CEE countries were not (yet) the EU members gave a basis to the position of ‘becoming European’ through the accession process. While actualizing in the process of the EU Eastern Enlargement, the subject position of becoming has a background in the eighteenth century, when Eastern Europe became one of the generalised Others in relation to whom Europe’s self-image was constructed (Neumann 1999, 143–60). Hence, it was simultaneously mapped as Europe but not Europe (Wolff 1994, 7; Moisio 2002, 98–9; Mälksoo 2006, 276).

The EU enlargement as a Europeanization of the CEE countries included elements from all three spheres of Europeanization – political, economic and cultural. As cultural Europeanization evolved in parallel with the EU Eastern Enlargement, in the time following the collapse of the Soviet Union, enlargement discussions often referred to cultural characteristics that the CEE countries were perceived to share with the EU countries, such as common history or religion. In the public discussion, the EU Eastern Enlargement was widely made sense as a process where the candidate countries transform their economic and political systems to become European whereas the EU and consequently also the notion of ‘European’ were taken as fixed and stable (see also Kaasik-Krogerus 2021). The past two decades have shown that rather than a stable ‘end point’, the EU membership means a continuity of transformation inside the Union. This has evoked controversial opinions and action in the CEE member countries. On the one hand, this has enabled the CEE countries to widen the scope of ‘being’ European by, for example, bringing the past under the Soviet regime and related heritage to the collective memories of Europe, including the EHL (Checkel and Katzenstein 2009, 3; see also Jones and Subotić 2011, 554). On the other hand, in the countries like Poland and Hungary, some have harshly criticized the Union in the context of the so-called refugee crises, claiming that its policy threatens the supposed ‘European values’. These examples demonstrate differing and changing perceptions of Europe.

This brings us back to the notion of belonging in terms of identification with the identity narratives as Europeanization from below. Indeed, various studies refer to the CEE citizens’ identification with ‘being’ European (e.g. Vihalemm 1997; Kirch and Kirch 2001; Góra and Mach 2017, 57) that can be interpreted as a sense of banal belonging to Europe. However, the research also shows that the ‘liminal’ position of the CEE countries has continued even after the EU enlargement (Mälksoo 2006, 2009; Velikonja 2011, 43–4; Ballinger 2017, 52; Komska 2018, 8–10). Thus, apart from ‘being’ European, also ‘becoming’ European has preserved its position in the enlarged EU, although in a latent form (Törnquist-Plewa and Stala 2011, 8–10). While criticism in the CEE countries was, and is, expressed towards being constructed as ‘liminal Europeans’, paradoxically the position of ‘becoming’ European is also self-ascribed and used, for example, in public discussion (see also Kaasik-Krogerus 2021).

We then understand identification with ‘becoming’ European as a struggle over belonging that aims at overcoming various controversies and hardships. Therefore, it is different from taken-for-granted banal belonging. In this context, Europeanization from below is entangled with both Europeanizing the CEE countries as a framework inherited from the accession period and engaging with the EHL as a prominent example of cultural Europeanization.

## Ethnography of Europeanization: field research at the EHL sites

For analyzing complex and contested issues like European identity and belonging from below in the framework of multifaceted Europeanization, a sophisticated complex of methods is needed. Thus, we use a methodological innovation called ‘ethnography of Europeanization’ (Lähdesmäki et al. 2020, 15–16), developed by our EUROHERIT research team for studying the EHL. The focus of this methodological approach is on the idea of Europe itself as an ongoing process and narrative constructed and governed by various actors at different levels, such as practitioners and policy-makers related to the EHL and cultural heritage in general. Our approach enables us to explore the constructions of identity and belonging in the context of power differences, inclusion, and exclusion in Europe by taking into account different people and multiple locations, levels and interconnected processes and thereby analyze the multiple layers of meanings (see also Marcus 1995; Falzon 2009). It helps to explore the meanings, which the participants and other actors in the EU’s cultural activities like the EHL give to Europe and in which ways their constructions engage with the EU’s cultural policy objectives, discourses and practices (Mäkinen 2022).

Due to the complex nature of Europeanization, there is no single location where one can study it. Therefore, the EUROHERIT team conducted mobile, multi-sited team ethnography (see Turunen et al. 2020) between summer 2017 and spring 2018 in 10 countries at 11 cultural heritage sites that have received the label (see Lähdesmäki et al. 2020). The sites selected for our fieldwork are Alcide De Gasperi House Museum, Italy; Archaeological Park Carnuntum, Austria; Camp Westerbork, The Netherlands; European District of Strasbourg, France; Franz Liszt Academy of Music, Hungary; Great Guild Hall, Estonia; Hambach Castle, Germany; Historic Gdańsk Shipyard, Poland; Mundaneum, Belgium; Robert Schuman’s House, France; and Sagres Promontory, Portugal.^1^ The data are analysed thoroughly in our previous publications (e.g. Lähdesmäki et al. 2020).

In this article, we use a specific part of the data gathered during our ethnographic fieldwork, namely semi-structured visitor and practitioner interviews.^2^ Altogether, we conducted interviews with cultural heritage practitioners working at these sites (*n* = 37) as well as with the visitors from 33 countries visiting the sites (*n* = 271). In this article, two data sets are used: the practitioner interviews conducted in three EHL sites located at the CEE countries (*n* = 10) and the interviews with the visitors from the CEE countries (*n* = 16) conducted at any of the 11 EHL sites. To avoid the juxtaposition of the ‘professionals’ and the ‘people’, the data are dealt with as one entity without making any systematic comparison between the practitioners’ and visitors’ answers (see also Kaasik-Krogerus 2021).

The first data set was collected through practitioner interviews conducted at the Historic Gdańsk Shipyard in Poland (four interviews), the Great Guild Hall in Tallinn, Estonia (three interviews) and the Franz Liszt Academy of Music in Budapest, Hungary (three interviews). The historic Gdańsk Shipyard was awarded the EHL in 2014 as a site with a key role in the collapse of the communist regime and a way towards democratic change in the CEE after 1989. In addition to the European Solidarity Centre (coded ESC) that hosts exhibitions of the site’s history, the site integrates other buildings and monuments, e.g. the historic Gate no. 2 where the leader of the Solidarity movement Lech Wałęsa made his speeches to the people and a Solidarity Square with the Monument to the Fallen Shipyard Workers of 1970. The main exhibition of the ESC presents the narrative of dialogue and peaceful change as ‘one of the foundations of modern Europe’ (Europejskie Centrum Solidarności n.d..).

The Great Guild Hall (coded GGH) is located in the Old Town of Tallinn. It was built in 1410 by the Great Guild, an association of German Hanseatic merchants in the medieval times, giving thus the visitors an impression of medieval Hanseatic architecture. The site was awarded the EHL in 2013 for the ‘intriguing story of the European “integration” in medieval times’ (EC European Commission 2013, 6) since the Guild played an important role in the framework of the Hansa for trade and cultural exchanges in medieval northern Europe. Since the 1950s, the building hosts the Estonian History Museum.

The Franz Liszt Academy of Music (coded LAM) was established in 1875 by the composer Franz Liszt himself. The site consists of the academy building hosting an international university of musical arts and a concert centre. In addition, the site integrates the Franz Liszt Memorial Museum and Research Centre, the Kodály Institute, and the Kodály Museum. The Academy was awarded the EHL in 2015 for nurturing, preserving, and developing ‘a living European cultural tradition’ and being ‘inherently international’ from the outset (EC European Commission 2015, 11).

The second data set consists of 16 interviews with the visitors from the CEE countries. Among these interviewees, there are 10 visitors from Poland, 2 visitors from Hungary, 1 visitor from the Czech Republic and 1 from Slovakia. In addition, one visitor has double nationality (Polish-German) and one has triple nationality (Hungarian-British-German). The low number of visitors from the CEE countries in comparison to all interviewed visitors can be explained by the fact that six of the selected 11 EHL sites happened to be situated in Belgium, France, Germany, Italy, and the Netherlands, and most of the interviewed visitors were citizens of these countries. However, our interviews at the Estonian heritage site did not include any Estonian visitors. The lack of local and national visitors at the Estonian and also the Hungarian site might be explained by the location of the Great Guild Hall in the middle of the touristic Tallinn Old Town that is included to the UNESCO World Heritage List and, respectively, site’s specific theme in Hungary – the Franz Liszt Academy of Music focuses on classical music. On the contrary, in the Historic Gdańsk Shipyard, almost half of the interviewed visitors were Polish.

The visitor interviews were conducted either individually or in pairs or small groups, whereas the practitioner interviews were conducted individually. The interviews were transcribed and translated into English, if needed. In the following analysis, we refer to the interviews with codes to ensure the anonymity of the interviewees. The codes starting with the letter P refer to the interviewed heritage practitioners, while the visitor codes start with the letter V.

We used qualitative content analysis to make sense of everyday Europeanization in the framework of the EHL. We started the analysis with a close reading of uses and meanings of the keywords related to ‘Europe’ and ‘European’. To scrutinize the construction and interplay of the positions of ‘being’, ‘becoming’ and belonging to Europe, we categorised the text on the basis of the subject positions of ‘becoming’, ‘challenging’ and ‘being’ European and identified static and processual aspects in each of the positions (e.g. Europe/the EU as fixed entities or dynamic, ongoing processes and constructions). These positions are not related to single actors or sites, but all interviews comprise a mixture of positions. The article focuses on the qualitative variety of the data and therefore no quantitative calculations were made as part of the analysis. Since our attention lies on the European scale and transnational angle, no systematic comparison on a national scale is made.

## Empirical analysis

Based on the theoretical framework presented above and the way the interviewees made sense of Europe and negotiated their national, institutional or personal belonging to Europe while engaging with the EHL sites, we constructed two analytically separated frameworks of Europeanization, namely the frameworks of struggle and banality. These frameworks emerge in the intersection of the EU Eastern enlargement and the cultural Europeanization, and we use them to analyse how interviewees elaborate their European belonging ‘from below’. Both frameworks mediate citizens’ perceptions of Europe and the European, and they are constituted by interviewees’ everyday encounters and experiences, including professional life (like work in the heritage sites) and leisure activities (like visiting the EHL heritage sites). In the framework of struggle, European belonging is elaborated from entangled subject positions of becoming European and challenging European that we have discussed in detail elsewhere (Kaasik-Krogerus 2021). The framework of banality is constructed predominantly around the subject position of being European. Since in the data the notions of identity and belonging were widely used as synonyms by the interviewees, apart from belonging also the term of identity figures in the analysis.

### Struggle over European belonging

The interviewees discussed the struggle over European belonging in close contact with the EHL sites and the cultural heritage displayed in them. Especially, the visitors of the European Solidarity Center (ESC) emphasized the importance of the struggle leading to the collapse and dissolution of the Soviet Bloc in the end of the 1980s in relation to their experience of Europe. The younger visitors pointed out that the everyday struggle of their parents and/or grandparents against the authoritarian rule provided the younger generation a ‘better world’ characterized by issues like freedom, liberty, solidarity and democracy (e.g. ESC V19; ESC V20; ESC V23). As one visitor said, ‘[i]t was all so small at first, it was Lech Walesa, just an electrician, and they came to work every day and did their things and then something was born in their hearts’ (ESC V23). According to another visitor, ‘[w]e used to be outside of Europe, but now we are in Europe, we are part of Europe’ (ESC V18). Although these people do not have a personal experience from the socialist period, they still feel engaged with these past events and consider this European heritage to be theirs (e.g. ESC V9; ESC V14; ESC V15). They see how these events have changed the world and played a key role in the process of ‘becoming European’ (Mälksoo 2009).

Moreover, some interviewees recognized the impact of this heritage of the CEE countries on the European integration and accordingly made sense of these EHL sites as significant part of European heritage. In the words of one visitor, ‘[t]he actions of the Poles influenced the events that took place in Europe’. (ESC V19; similar ESC V20; ESC V22; ESC V23; ESC V24; ESC P1; ESC P4). Such accounts were used for broadening the notion of what is ‘Europe’ and ‘European heritage’. According to one practitioner, the past events in Poland should be seen as equal to events widely recognized as crucial for the European integration:

European integration is not something that is only connected to Roman treaties or to Western European consolidation after the Second World War. It is something that is connected also to the process of civic emancipation here, and that Poland is participating in creating modern democracy now for hundreds of years

(ESC P3).

Highlighting the contributions that the developments in the CEE countries have made to the European integration can be interpreted as challenging the existing narratives of Europe and European integration as too narrow. Alongside with this understanding, an ongoing liminality of the CEE countries and citizens was communicated in the interviews (for liminality see Mälksoo 2006, 2009; Ballinger 2017, 52; Komska 2018, 8–10). ‘Not (yet) Europeans’ was one way of expressing this liminality. Getting used to, accepting and appreciating a diversity of people, languages, minorities, nations, cultures, regions and understandings as a feature characteristic to Europe, was seen to be a remarkable challenge for the CEE citizens (GGH P1; similar ESC V18; LAM P2; LAM P3) and this can be interpreted as a sign of a liminal position.

The state of continuously seeking for ‘European’ recognition was also presented in the interviews as a basis for liminality. Indicating an ongoing process of ‘becoming European’, one practitioner explains how being awarded the EHL as a European label is indeed important for people’s identity:

It is really important for Estonians to belong to Europe. And this whole heritage label also kind of clearly has this result that we belong to Europe, right, our museum has got it

(GGH P1).

The data shows that diverse obstacles faced in the struggle over belonging to Europe may turn the process of belonging towards a process of non-belonging. According to one visitor, European identity means that people can rely on the law and trust that everyone keeps it, whereas at the moment that is not the case (CAP V10). The situation is impacted by political developments like ‘two-speed Europe’ (LAM P1) and the political leaderships of Poland and Hungary who are seen by various interviewees as not Europe-minded (e.g. ESC V24; ESC P3, ESC P4). An interviewee elaborates that ‘[w]e belong to Europe, and I think it’s especially now in Hungary a very crucial question, because our current government seems to be questioning this, which I am personally very angry with‘ (LAM P1). This interviewee explains that joining in the EU, which she terms ‘re-inclusion’ of Hungary in Europe, was a great moment that still means a lot to her. Contemporary developments in Hungary challenge this memory and accordingly the notion of European belonging. Similarly, another practitioner presents a future vision concerning her professional life: ‘if we have more and more nationalism in different countries, so maybe one day the people throw away the European label and they start to talk only about national label’ (ESC P1).

At the same time, several practitioners claimed that at least in principle the EHL fosters the idea of European heritage and hence has a potential to facilitate a sense of unity and belonging in Europe (ESC P2; GGH P1; GGH P3; LAM P2; LAM P3). The EHL thus provides a context for reflecting belonging to Europe and the EU. In these reflections, the EU can play ‘an important symbolic role […] allowing national states […] to escape the shadow of the past and to embrace a new understanding of what it is to be German, Romanian or Hungarian in an EU context’ (Cram 2009b, 105).

One context in which the interviewees elaborated the struggle between belonging and non-belonging was mobility and migration. For instance, in the framework of higher education and related heritage, mobility was seen by some interviewees as a positive phenomenon that helps to strengthen people’s sense of belonging to Europe (LAM P1; LAM P2). Feeling European was connected to traveling and the ‘freedom to move anywhere I want in Europe’ (ESC V25; similar also ESC V24; ESC P1). The interviewees recognized the EU’s role for facilitating freedom of mobility, which can strengthen the functional aspect of their European identification (Cram 2012, 74–76): everyday experiences of travelling can support ‘becoming European’. Respectively, there were single interviewees who elaborated their rather weak sense of belonging to Europe in the context of not traveling much outside their home country (ESC P15).

On the other hand, the meanings devoted to migration differed from the ones attached to previous kind of mobility. Some interviewees raised concerns about how migration from ‘not only the European nations’ was a ‘big issue’ that has to be ’solved’ in the EU (LAM P2; similar ESC P4; LAM P1; LAM P3). In the words of one visitor, migration or more specifically refugees seeking asylum in the EU should not be let to ‘break the inside connection’ (ESC V18). Hence, based on the interviews, ‘eligible’ mobility discussed above can strengthen the European sense of belonging, whereas ‘problematic’ migration is seen to undermine it. However, migration was also dealt with through a more inclusive, yet occasionally patronizing and Eurocentric approach. Some interviewees emphasized a need not to ‘leave the refugees alone’ or ‘outside’ but to ‘introduce them Europe and European culture’ as well as to ‘include them to European culture’ (GGH P2; similar ESC P1; ESC P2).

### Banal belonging to Europe

While the struggle over belonging was constituted by tackling the hardships and overcoming various controversies, in the second framework European belonging appears as a taken-for-granted state of order that can be conceptualized as banal, following Billig (1995) and Cram (2009b, 2012).

When engaging with the EHL sites, the interviewees created a strong link between the national and European scales. Some interviewees did this by discussing the significance of the transnational and cross-border dimension of European belonging, pointing out that people can be ‘partially German and then Russian and then Poles and then Italian’ (ESC P2). The practitioners of the Liszt Museum explicate the importance of transnationality in the background of being European based on Franz Liszt as a remarkable part of European cultural heritage and therefore a potential ‘role model’:

Franz Liszt […] who was a true Europeaner, a very cosmopolitan person who believed in Europe, and he traveled a lot. He was born in Austria, but Hungarian, but his first language was German, but the best language he spoke was French, so he was a very European traveler, and he spent a lot of time in Italy and in Germany

(LAM P2).

Especially in the visitor interviews. this strong link between the national and European scales made explicit the banality of national belonging (Billig 1995). The interviewees took it for granted that their countries belonged to Europe and that national heritage was part of European heritage. Some interviewees felt that their own home country’s joining the European Union strengthened their European belonging (e.g. GGH P2). As the interviewees said, ‘I feel Polish, so I have to feel European’ (ESC V18) and ‘[m]y country [Slovakia] is European’ (CAP V1; similar also ESC V14; ESC V15; ESC V19; ESC V20). Frequent micro-level engagement with EU-related practices and representations, such as passports, driving licences, legislation, and EU ﬂags, may ‘remind citizens of their involvement in the larger EU system’ and effect ways of identification that are not necessary ‘passionate or heroic but mundane, even banal’ (Cram 2009b, 104–105).

This ‘Russian doll’ model where belonging to different scales – from the smallest to the largest – is seen as a natural order of things (Lähdesmäki et al. 2020) was communicated by various interviewees (ESC P2; ESC P4; LAM P1; LAM P3). The interviews present the relation of national and European identification as synergistic, which implies a multidirectional conception of Europeanization (Cram 2009b, 106). Some interviewees built a hierarchy of their belonging, seeing national belonging as more intimate and therefore also stronger than belonging to Europe:

So, I feel European, but above all I feel as a Pole. For me, both of these affiliations that define me as a human being are very important, but I do not hide that it is more important to be a Pole, and after that a European

(ESC V22).

Some also brought to the fore the importance of the personal scale, stressing that people belong to Europe as individuals. According to one interviewee, ‘I am part of Europe, not only my country, but also me … as a person’ (ESC P1; similar also ESC P2; ESC V23). As another captured it, ‘people are Europe’ and no matter what people do ‘we are being Europe’ (LAM P2; similar also ESC P1; ESC P4; GGH P1).

The data included single readings of European belonging as (too) narrow for the interviewees. According to one interviewee, ‘I want to be European, but it’s also too small for me emotionally’ (ESC P3). Another claimed that she felt European but even more as a citizen of the world (ESC V19). This can be interpreted as preferring a broader viewpoint to the strict divisions between scales.

In their contemplations, the interviewees made sense of what is characteristic to (belonging to) Europe as well as to ‘European’ heritage. Europe was seen as a ‘community of idea’ (ESC P1) constituted by various cultures, religions, nations, countries as well as certain values and characteristics like freedom, solidarity, dialogue, trends of philosophy and architecture (CAP V1; CAP V18; ESC V23; ESC V24; ESC P2; ESC P4; GGH P2; GGH P3; LAM P1; LAM P2). In the words of one interviewee, ‘our heritage, our cultural experience, historical experience is a sense of being European’ (ESC P2; similar also GGH P2; GGH P3). Some interviewees emphasised the importance of the rather exclusive characteristics of Europe. Some of them pointed out things like common roots, common ground of lifestyle and traditions as well as the role of Christian religion and background (CAP V10; LAM P1; LAM P2; LAM P3).

As we mentioned earlier, the EU accession of the CEE countries was legitimized by depicting the candidates as ‘being’ European indeed based on shared history, memory, culture, and values. For the CEE countries, ‘the notion of coming “back to Europe”, or of a shared history interrupted, was a signiﬁcant element of the accession discourse’ (Cram 2009b, 106). These also being central tools in the EU’s identity politics, the EHL aims to produce an idea of a European cultural heritage, which has values as its core contents. A ritualistic repetition of values and principles with a strong European framing is typical also for the EU discourse in general and can be interpreted as a banal way of producing identity (Mäkinen 2019).

Interviewees also highlighted the importance of civic aspects by stating that Europe is more than a sum of the nation states. According to this understanding, Europe is not restricted to Christian background or common cultural heritage. Thus, also other people than those ‘born here’ can be Europeans, since an open-minded attitude is a key feature of Europeanness: ‘[s]o, everybody who wants to be part of a democratic pluralistic tradition’ (ESC P3). Similarly, another practitioner claims that ‘if you believe in the same idea, it doesn’t matter, where you live, you are still European’ (ESC P1). A more concrete elaboration about this ‘idea’ is mediated in the following quote:

I think being a European is primarily about identifying with this place and with those values. And these values can be, for example, openness to another person and openness to diversity. […] and we talk about diversity, we talk about people of different views, we talk about people of different colour, different race, nationality, culture, religion, sexual orientation. And I think that this is primarily written in the European identity

(ESC V20).

The interviewees expressed an understanding that although it is not that easy to name and depict the characteristics and dimensions of what constitutes Europe, ‘the European’ and related heritage, people still recognize them. According to one practitioner, it is easy to take for granted that heritage in Europe is European heritage and European people in general identify this heritage as such even though it is not unified (GGH P1; similar also ESC P4; LAM P2). Indeed, reminders of national or European identity may be so mundane that they are ‘not consciously registered as reminding’ (Billig 1995, 8). As Cram (2009b, 104–105) writes, ‘[f]or EU citizens, identiﬁcation may […] be based on daily low-level engagement in unremarkable ways’.

Some interviewees used a comparative approach including the notion of what is *not* European to make sense of European belonging. To articulate what ‘the European’ is about, they made distinction between European characteristics on the one hand and Asian, American and African characteristics on the other (e.g. GGH P1; LAM P1; LAM P2). Sometimes this distinction-making leads towards a Eurocentric approach where ‘Europeanness’ was connected to a certain quality level. Terms like ‘European quality’, ‘highest European standards’ or ‘being on European level’ mediated an understanding of ‘the European’ as not just different or having specific heritage but as a quality label and therefore exemplar for those who supposedly are not on that level (LAM P1; LAM P3). Unlike in the framework of struggle over belonging that is constituted by the notion of liminality and the subject position of becoming European, here the interviewees identified either themselves and/or their institutions as ‘European’/part of European cultural heritage and hence representatives and examples of ‘highest European level’.

## Discussion and conclusions

Europeanization is a contradictory process constituted by the negotiations over the meanings and boundaries of Europe (see also Flockhart 2010). It is always in the making without a clear ‘end result’. As the article shows, ethnography of Europeanization is a useful method for producing a nuanced understanding of the negotiations over everyday Europeanization and being, becoming and belonging to Europe. It enables scrutinizing the complex dynamics of identification simultaneously from below and above. In future studies, it can be used for analyzing entanglement and interaction of actors from different locations, scales, and positions in various dynamic processes, including multilevel governance.

The analysis based on the ethnography of Europeanization offered a transnational angle to European identity and belonging by focusing on heritage sites located all over the EU and the visitors coming from various CEE countries. The interviewees engaging with the EHL sites discussed mostly political and cultural Europeanization, while economic Europeanization was referred to mainly implicitly through their contemplations concerning mobility. The CEE citizens’ experiences of ‘Europe’ and ‘European heritage’ indicate transnational aspects of belonging as well as a multifaceted process of Europeanization constituted by the frameworks of struggle and banality.

The struggle for becoming European was discussed in the context of the struggle for freedom, liberty, solidarity, and democracy and against the authoritarian rule in the CEE countries. The collapse of the Soviet bloc was seen as an opportunity to ‘return’ to Europe for these countries.

Another central context for the discussions on the struggle for becoming European was the EU and the accession process of the CEE countries. The interviewees highlighted the need to overcome the liminal position of the CEE countries by recognizing the contributions of the CEE countries for the integration, thereby challenging the prevailing notions of ‘Europe’ and ‘European heritage’. Simultaneously, the interviews referred to an ongoing sought for ‘European’ recognition. This can be interpreted as an indication of a liminal position of being European but not quite. The continuous struggle between belonging and non-belonging was also addressed in the context of mobility: mobility was experienced as facilitating the European belonging. In addition, belonging was deliberated in relation to ‘others’ in terms of migrants from countries outside of Europe, perceived as outsiders from the perspective of European heritage. Hence, the framework of struggle in our analysis shows how the struggle for Europeanization is entangled with struggle against it and how non-belonging can be part of belonging.

Banal belonging to Europe involves interviewees’ experiences that Europe, Europeanness and European cultural heritage are something self-evident. Banal Europeanism (Cram 2009a, 2012) includes everyday routine practices and daily encounters with EU symbols that may bring the EU ‘closer to the people’ and reinforce (unconscious) identification with Europe. It was often mediated by the national level: the EU membership of the interviewees’ home country offered a basis for feeling belonging to Europe. While this European belonging was taken for granted, Europe, its heritage and a sense of Europeanness were nevertheless experienced as something fuzzy. The interviewees attached several types of elements to the notions of Europe and Europeanness, such as values, diversity, and historical background. They also elaborated these notions through comparisons with the ‘non-European’ with both inclusive and exclusive connotations. Banal, contingent and contextual processes that emphasise complementary interests may lead to the ‘normalisation’ of the EU as a legitimate political authority. In Billig’s (1995) terms, the EU has become enhabited as individuals ‘forget to remember’ that the current situation is not how things always were (Cram 2009a).

## Notes

1. The project has been reviewed and approved by the Human Sciences Ethics Committee of the University of Jyväskylä. The ethical review of the Committee is based on the guidelines of the Finnish Advisory Board on Research Integrity.

2. In addition to these interviews, our data gathering encompassed interviews with key EU heritage officials and a representative of the EHL selection panel (*n* = 7), photos taken by the interviewed visitors at the EHL sites; a survey of national coordinators of the EHL in the selected ten countries; and informal discussions with guides and various stakeholders of the sites. The data also included multifaceted observation of these sites (e.g. their exhibition narratives) collected as videos, catalogues, photographs, audio recordings, and notes in field journals and site memos, as well as diverse (multimodal) textual material, such as websites, promotional and educational materials. We asked all 38 EHL sites which were labelled before 2018 to share their EHL applications with us and received 16 applications. Our data also included 8,299 official EU documents based on a search for the word ‘heritage’ in the EUR-Lex database.
